# Rethinking public health campaigns in the COVID-19 era: a call to improve effectiveness, equity and impact

**DOI:** 10.1136/bmjgh-2021-006397

**Published:** 2021-11-26

**Authors:** Hamid Jafari, Kristin N Saarlas, W William Schluter, Marcos Espinal, Kashef Ijaz, Christopher Gregory, Scott Filler, Chris Wolff, L Kendall Krause, Katherine O'Brien, Luwei Pearson, Anuradha Gupta, Maria Rebollo Polo, Faisal Shuaib

**Affiliations:** 1 Polio Eradication, WHO Regional Office for the Eastern Mediterranean, Amman, Jordan; 2 Health Campaign Effectiveness Program, The Task Force for Global Health, Decatur, Georgia, USA; 3 Center for Global Health, Centers for Disease Control and Prevention, Atlanta, Georgia, USA; 4 Communicable Diseases and Environmental Determinants, Pan American Health Organization, Washington, District of Columbia, USA; 5 Health Programs, The Carter Center, Atlanta, Georgia, USA; 6 Immunization Unit, UNICEF, New York, New York, USA; 7 Malaria Team, The Global Fund to Fight AIDS, Tuberculosis and Malaria, Geneva, Switzerland; 8 Bill & Melinda Gates Foundation, Seattle, Washington, USA; 9 Immunization, Vaccines and Biologicals, WHO, Geneva, Switzerland; 10 Health Programme Division, UNICEF, New York, New York, USA; 11 GAVI Alliance, Geneva, Switzerland; 12 ESPEN, WHO Regional Office for Africa, Brazzaville, Congo; 13 National Primary Health Care Development Agency (NPHCDA), Abuja, Nigeria

**Keywords:** COVID-19, health policy, health systems, immunisation, infections, diseases, disorders, injuries

**Summary box**

- Health campaigns are time-bound, intermittent activities that address specific epidemiological challenges, expediently fill delivery gaps or provide surge coverage for health interventions.
- They can be used to prevent or respond to disease outbreaks, control or eliminate targeted diseases as a public health problem, eradicate a disease altogether or achieve other health goals.
- In 2020, more than 530 health campaigns were planned for 26 different interventions representing 13 diseases and 105 countries.
- Due to the COVID-19 pandemic, by the end of last year, about half of planned campaigns were postponed, cancelled, or suspended leaving hundreds of millions of children and families at risk of vaccine-preventable diseases, tropical diseases and malnutrition.
- So far in 2021, more than 280 campaigns have been cancelled or delayed.
- As health campaigns restart and catch up on the delivery of missed drugs, vaccines and nutritional supplements, and countries roll out COVID-19 vaccines, there is an opportunity to rethink the way we plan and implement campaigns to improve their effectiveness and impact.

**Summary box**

- The Health Campaign Effectiveness Coalition was formed in early 2020 with a vision that country-led health systems use a strategic balance of targeted health campaigns in concert with routine and primary healthcare services to achieve and sustain health-related development goals for all people.
- The coalition’s purpose is to foster shared learning across different campaign programmes used to deliver immunisations, eradicate polio, prevent and treat neglected tropical diseases and malaria, and provide vitamin A supplementation.
- The coalition supports countries to develop and research innovative campaign delivery approaches; accelerates the dissemination and adoption of promising practices; and works to strengthen countries' capacity to engage with global campaign partners.
- As members of the coalition’s Leadership Team and representatives of various global, country and donor organisations for health campaigns, we intend to leverage our collective expertise to support collaborative approaches that ensure these essential health interventions reach all those in need.
- We encourage all campaign stakeholders to explore and support opportunities for joint planning and implementation, sharing of supply chains and logistics, information systems, and demand creation and community-based engagement strategies.

## The effect of COVID-19 on critical health programmes

By September 2021, the COVID-19 pandemic has resulted in more than 4.7 million reported deaths globally and caused sweeping disruptions to all aspects of health systems.^1^ In addition to direct effects on morbidity and mortality, the pandemic has hindered the ability to access and provide routine healthcare services in all countries^2^ and worsened the plight of conflict-affected communities and hard-to-access populations. The pandemic has also disrupted delivery of and access to critical public health campaigns that provide essential childhood vaccines; deliver nutritional supplements; and control, eliminate, or eradicate a variety of debilitating neglected tropical diseases (NTDs). Although campaigns for delivering insecticide-treated bednets (ITNs) for malaria prevention and vaccines for polio and some other diseases successfully adapted approaches to safely continue or resume services, data from the COVID-19 Impact Tracker indicate that, as of 31 December 2020, more than half of the 530 campaigns planned to occur in 2020 were suspended, postponed or cancelled with similar delays already in 2021.^3 4^ Countries’ health systems have also been affected by the devastating economic impact of the pandemic.^5^

The disruption of immunisation campaigns alone has left at least 228 million children across more than 50 countries at risk of diseases such as measles, yellow fever and polio.^6^ Many NTDs treated with mass drug administration campaigns and activities have also been disrupted at a time when NTD programmes were progressing toward ambitious goals for 2030.^7^ Outbreaks of vaccine-derived poliovirus expanded in several countries. Countries could experience especially fast resurgence of these diseases if they are unable to safely resume activities in the near future.^8^

The daunting challenge now facing the countries and the broader global health community is how to safely and expeditiously implement essential health campaigns, strengthen primary healthcare (PHC) systems and simultaneously introduce COVID-19 vaccines worldwide. This challenge will be acutely felt by community health workers (CHWs) and local health systems that are understaffed and have limited resources to coordinate multiple campaigns. There is an added cost and burden to campaign implementation during the pandemic due to the need for additional personal protective equipment, such as masks and gloves, training of health workers and efforts to ensure the trust and engagement of communities in campaign delivery approaches.^9^

## An opportunity to rethink how campaigns can better align with PHC strategies and reach unreached communities

If there is any upside (or silver lining) to disruption from the COVID-19 pandemic, it is that it has created a sense of urgency to rethink the way we plan and implement campaigns to increase their effectiveness, efficiency, equity and alignment with national PHC strategies.

Several of our respective organisations have already recognised this need and opportunity. The WHO, the Global Polio Eradication Initiative, UNICEF and Gavi the Vaccine Alliance have each recently released guidance highlighting approaches that shift away from single-disease programmes to a Health-in-All Policy that supports integrated approaches and improves coordination and collaboration among programmes.^10–16^ They have also called for emphasis to be placed on reaching zero dose children and marginalised communities who have not been reached previously by campaigns.

Historically, health campaign programmes have operated independently from each other, often with limited coordination across programmes and with country health systems. This has resulted in inefficiencies and inequities that can burden healthcare workers and limit the potential impact of these important health interventions. To address this problem, the Health Campaign Effectiveness (HCE) Coalition was formed (the HCE Coalition is funded by a grant from the Bill & Melinda Gates Foundation to the Task Force for Global Health, a 501(c)3 non-governmental organisation based in Atlanta, Georgia, USA) in early 2020, with the purpose to foster shared learning across different campaign programmes, support countries and partners to undertake implementation research on effective campaign approaches, accelerate the adoption of promising practices and strengthen country capacity to engage with global campaign partners.^17^ The HCE Coalition brings together country leaders, campaign managers, implementing partners, donors and researchers from across multiple health campaign programmes. Thus far, more than 920 individuals have engaged with the coalition representing NTDs, polio and other vaccine-preventable diseases, malaria and vitamin A supplementation programmes from over 50 countries and 120 organisations. Furthermore, the goals of the coalition are endorsed by key global, regional and national stakeholders including WHO, Pan American Health Organization (PAHO), UNICEF, The Global Fund, Gavi the Vaccine Alliance and the Centers for Disease Control and Prevention (CDC). As members of the coalition’s Leadership Team, we believe the coalition can serve as a catalyst and a platform for the coordination and integration that WHO and others have called for as a means to strengthen PHC systems while ensuring campaign-based delivery meets population needs. We encourage others to actively participate in the coalition and recommend we collectively work to support the following actions.

## A call to action: recommendations to improve campaign effectiveness, equity and impact

1. **Strengthen collaboration and shared action among the different programmatic areas and campaign platforms**. This will enable the identification of opportunities to improve campaign effectiveness, equity and impact. Examples of collaborative activities include: sharing micro-plans, COVID-19 safety protocols and local knowledge; coordinating supply chains; engaging local communities, including CHW training and social behaviour change messaging; and sharing and integrating data from health information and surveillance systems. We should also engage with sectors outside of health (eg, education, private industry and finance) to identify innovative and effective delivery approaches.
2. **Engage communities and local leaders in solutions**. Community engagement, acceptance and partnership are critical to building community trust and sustaining health gains, but programmatic practices are often inflexible and disempowering to those at the local level. Implementing risk communication and community engagement strategies will identify local solutions while mitigating misinformation and mistrust.^18^ Champions at all levels (eg, community, local, regional, national) should be encouraged to come forward with innovative ideas that can improve access and help achieve shared health goals.
3. **Support and test integrated and collaborative campaign service delivery approaches**, especially in communities affected by multiple diseases, where feasible and appropriate. This requires supporting countries and partner organisations to conduct implementation research on community and health worker acceptance of integrated (from collaborative to co-delivery) approaches,^19^ digitise campaigns, examine incentive structures and conduct economic analyses, as well as to develop risk communication approaches to manage misinformation that might undermine integration efforts. We need to work together to identify evidence-based practices and reach consensus on strategies for campaign delivery that also strengthen the PHC system. The HCE Coalition is currently supporting 18 country studies whose findings will be shared on the HCE website at http://campaigneffectiveness.org.
4. **Adopt funding approaches and policies that enable and incentivise countries to use campaigns more strategically, thus reducing inequities and competition between campaigns and PHC services**. There is a need to review the ways that payments to CHWs and community drug distributors are structured across different campaigns and minimise unnecessary variation. Differences in programme funding by donors to community campaign health workers have been a barrier to collaborative and integrated approaches and can lead to disincentives and, by extension, inequities between programmes. A fair and sustainable funding approach, which should include incentives for reaching previously missed, high-risk populations, is needed. Incentives for routine services based on results instead of campaigns incentives alone are needed in countries planning multiple and repeated campaigns.
5. **Examine opportunities to coordinate and build on current health campaign platforms and country expertise during delivery of COVID-19 vaccines**. The pressure to quickly deliver COVID-19 vaccines is intense; however, we should use this opportunity to also reach populations in need of other vaccines, drugs to treat NTDs, malaria ITNs and nutritional interventions. We cannot risk further delays in delivering these essential services and must optimise limited resources by better coordinating our health programmes.

## Conclusion

We are at a unique moment. Although there is growing pressure to implement improved approaches and lessons to be learnt from past and current campaigns, it will take a coordinated effort to make the programmatic and policy shifts that will enable us to achieve shared health goals. As members of the HCE Leadership Team, we call on all health campaign stakeholders, including Ministries of Health, local and international non-governmental and civil society organisations to partner with us to identify new solutions and serve as early adopters of innovations in campaign delivery. We encourage donors, academic institutions and partner organisations to support and share implementation research findings and promising practices so we collectively build the evidence base for more effective and equitable campaigns. We must seize this moment to work together to ensure that critical health interventions, including COVID-19 vaccines and prevention strategies, safely and effectively reach all populations in need.

## Data Availability

No data are available.
